# The adverse effect of ambient temperature on respiratory deaths in a high population density area: the case of Malta

**DOI:** 10.1186/s12931-022-02218-z

**Published:** 2022-10-31

**Authors:** Shafkat Jahan, John Paul Cauchi, Charles Galdies, Kathleen England, Darren Wraith

**Affiliations:** 1 School of Public Health and Social Work, Faculty of Health, Queensland University of Technology, Victoria Park Rd, Kelvin Grove, QLD 4059 Australia; 2 Institute of Earth Systems, University of Malta, Msida, MSD 2080 Malta; 3 Directorate for Health Information and Research, Ministry for Health, Tal-Pietà, Malta

**Keywords:** Respiratory mortality, Ambient temperatures, Cold effect, Distributed lag nonlinear model, Time varying effects

## Abstract

**Background:**

The effect of ambient temperature on respiratory mortality has been consistently observed throughout the world under different climate change scenarios. Countries experiencing greater inter–annual variability in winter temperatures (and may not be lowest winter temperatures) have greater excess winter mortality compared to countries with colder winters. This study investigates the association between temperature and respiratory deaths in Malta which has one of the highest population densities in the world with a climate that is very hot in summer and mild in winter.

**Methods:**

Daily number of respiratory deaths (7679 deaths) and meteorological data (daily average temperature, daily average humidity) were obtained from January 1992 to December 2017. The hot and cold effects were estimated at different temperatures using distributed lag non-linear models (DLNM) with a Poisson distribution, controlling for time trend, relative humidity and holidays. The reference temperature (MMT) for the minimum response-exposure relationship was estimated and the harvesting effects of daily temperature (0–27 lag days) were investigated for daily respiratory mortality. Effects were also explored for different age groups, gender and time periods.

**Results:**

Cooler temperatures (8–15 °C) were significantly related to higher respiratory mortality. At 8.9 °C (1st percentile), the overall effect of daily mean temperature was related to respiratory deaths (RR 2.24, 95%CI 1.10–4.54). These effects were also found for males (95%CI 1.06–7.77) and males across different age groups (Males Over 65 years: RR 4.85, 95%CI 2.02–11.63 vs Males between 16 and 64 years: RR 5.00, 95%CI 2.08–12.03) but not for females. Interestingly, colder temperatures were related to respiratory deaths in the earliest time period (1992–2000), however, no strong cold effect was observed for later periods (2000–2017). In contrast, no heat effect was observed during the study period and across other groups.

**Conclusions:**

The higher risk for cold-related respiratory mortality observed in this study could be due to greater inter-annual variability in winter temperatures which needs further exploration after adjusting for potential physical and socio-demographic attributes. The study provides useful evidence for policymakers to improve local warning systems, adaptation, and intervention strategies to reduce the impact of cold temperatures.

**Supplementary Information:**

The online version contains supplementary material available at 10.1186/s12931-022-02218-z.

## Background

Respiratory diseases are the third leading cause of death in European countries, accounting for 8% of all deaths in 2016 [1]. Evidence on the role that meteorological variables play is well established with an extensive literature on the relationship between ambient temperature and its effect on respiratory mortality in various cities and countries around the world [2–6]. The impact of climate change on respiratory illnesses has attracted renewed interest due to sudden changes in temperature from average levels and increased frequency of extreme weather events [6–8].

Previous studies have used different methodological approaches to examine the exposure-lag response relationship between ambient temperatures and respiratory mortality in populations with different geographic locations, climates and different socio-demographic characteristics [2–4, 7–9]. Using different temperature measures (mean temperature, maximum and minimum temperature, temperature variability, apparent temperature) and statistical analysis (i.e., Distributed lag non-linear models, Generalised linear models, Generalised additive models), these studies established a J or U-shaped relationship between ambient temperature and respiratory deaths with a lower number of deaths at moderate temperatures, rising progressively with higher and lower temperatures.

Gasparrini et al. [10] examined the cold and heat-related mortality in 386 countries and reported that the highest mortality risk was related to moderately cold temperatures with an overall estimate of 6·66% (95%CI 6·41–6·86) compared to extreme cold and hot temperatures. The temperature percentiles for minimum mortality vary significantly in countries with tropical climates (60th percentile) to countries with the temperate and Mediterranean climates (80–90th percentiles). The higher attributable risk was reported for temperate countries (i.e., China, Italy, and Japan) compared to tropical countries (i.e., Thailand, Brazil and Sweden).

Studies from European countries have reported effects of extreme temperatures (higher and lower) and extreme weather events (heat waves and cold spells) on mortality due to different respiratory diseases including Chronic obstructive pulmonary disease and Asthma, particularly in elderly populations [10–17]. Analities et al. [11] reported an inverse relationship between apparent temperature and respiratory mortality during the winter season and the effects were higher for warmer Mediterranean cities compared to colder North-central cities. D'Ippoliti et al. [13] also assessed heatwave effects on mortality in 9 European cities and reported the highest mortality for respiratory diseases among women aged 75–84 years in warmer Mediterranean cities (61.3%, 90%CI 44.3–80.4%) compared to colder north Continental cities (21.4% 90%CI 12.9–30.6%).

There could be several reasons for an increase in deaths relating to respiratory diseases during extreme temperatures and extreme weather events. Cold temperatures may reduce natural defensive mechanisms of the upper respiratory tract and suppress an immune response to infections [14]. Elderly, small children, socially isolated individuals, people with less adaptive and coping strategies are more susceptible to pulmonary problems due to broncho-constriction caused by breathing cold air [18]. Influenza during winter seasons can be also fatal for elderly people especially with other underlying conditions. Elderly patients with pre-existing respiratory problems (Chronic obstructive pulmonary diseases, Asthma) are more susceptible to heat or cold effects due to their limited thermoregulation and other comorbid conditions.

With climate temperatures set to increase on average globally, the occurrence of extreme weather events is also set to increase in many regions around the world [10, 19, 20]. The Mediterranean basin is no exception with hot spells set to become more frequent in the coming decades, especially over the summer months. The recent ‘Lucifer’ heatwave that occurred over Southern Europe in August 2017 is one such example, with the World Weather Attribution group (WWA) saying that it was made four times more likely to occur due to climate change [21].

Malta is an archipelago situated to the south of Sicily (southern Europe) and shares a Mediterranean climate. Galdies et al. [22] found a local warming trend in the annual maximum temperature by 0.09 °C per annum for the period 1967–2013, accompanied by a positive trend of + 0.2 °C per decade in the mean annual air temperature for the period 1951–2010. Malta is expected to experience a moderate effect of climate change with a projected rise in annual average temperature in the range of 0.53–1.32 °C by 2030 [23, 24] with negative impacts on a number of important sectors [25]. Akerlof et al. [26] conducted a survey on community perceptions of climate change impacts and reported that 91% of Maltese people believe that climate change can cause respiratory problems.

In contrast with other studies, Malta provides a unique and contrasting insight into the effect of ambient temperature on respiratory mortality due to a mostly urban island landscape, higher population density and typical Mediterranean climate with a very hot summer and mild winter. Studies from major provinces of China show that higher respiratory mortality risk in elderly people might be due to higher population density which increases the risk of influenza transmission [27, 28].

During the last decade, significant emphasis has been given to temperature/ climate change adaptation strategies (i.e., air conditioning and heater use) to mitigate the effects of climate, particularly in the hotter months. From a public health perspective, Malta’s Health Promotion and Disease Prevention Directorate frequently issue hot weather warnings in the event of extreme temperature scenarios such as heatwaves.

To the best of the authors’ knowledge, no studies have been found from Malta examining the potential impact of ambient temperature and extreme weather events on respiratory mortality. England et al. [29] investigated the relationship between ambient temperature and all-cause mortality in Malta and reported that the daily mortality rate was higher during winter (18.07/100,000) at 11.57 °C compared to summer (12.46/100,000) at 29.93 °C during 1992–2005. While the mortality rate per degree increase (above 27 degrees Celsius) in daily apparent temperature was higher during summer compared to the per degree fall in temperature (from 27 °C) during winter (3.03% vs 2.52%), the number of deaths at a very low temperature was greater when compared to summer. However, the results were not specific to the cause of mortality, so the adverse effect of temperature on respiratory mortality is unknown. Moreover, lag effects (0–15 days) of temperature on all-cause mortality were not clearly examined or discussed. Improved and cause-specific statistical evidence is required to inform policymakers and improve current adoption and mitigation strategies.

We conducted this study to examine the impact of ambient temperature on respiratory deaths in the Maltese population from 1992 to 2017. This long time period allows greater confidence to be established for relationships and also to assess the effects of any changes in these relationships over time. The study utilised time series regression models and analysed short term and delayed effects of ambient temperature on respiratory mortalities. Additionally, subgroup analysis was conducted by age groups, gender and time periods to examine effects of temperature extremes on vulnerable populations.

## Methods

### Study settings

Malta is a small island state in the Central Mediterranean, lying at approximately 35°N and 14°E. It includes two main islands—Malta and Gozo, with a combined population of around 440,433 in 2016 [30]. Its total area is 316 km^2^ thus giving it a population density of 1,393 persons/km^2^. This makes it one of the most densely populated places on Earth, and the most densely populated country in the European Union [31]. It has a semiarid Mediterranean climate, with hot, dry summers and mild, wet winters, and has a high life expectancy of 79.7 years for males and 83.7 for females [32].

The temperature is considered to be very stable, the annual mean being 18 °C and monthly averages ranging from 12 °C in January to 31 °C in July–August. The relative humidity rarely falls below 40% and is often high. Summers are warm, dry and very sunny. With barely any rain, July and August are Malta’s hottest months with daytime temperatures usually above 30 °C. Summer temperatures can feel very high due to the high humidity and this is especially so in August and September, with often high humidity at night [24].

### Mortality data

Daily mortality data (*n* = 7679) due to respiratory diseases was obtained from Malta’s mortality register for the years 1992–2017, with permission from the Directorate for Health Information and Research in Malta [33]. The data included the age at death, gender and was limited to only residents of the country. The supplied data has no missing values and age and gender were categorised into these groups: male, female, male 0 to ≤ 15 years, male between15 and < 65 and male over 65 years +, female 0 to ≤ 15 years, female between 16 and < 65 and female over 65 years +, respectively.

### Meteorological data

Weather data collected from Malta’s sole Climatological Station situated at Luqa were obtained from the global surface archives of WeatherGraphics.com in the form of 3-hourly machine coded SYNOP data (WMO FM 14). The decoded dataset consisted of the time stamp, air temperature, dewpoint temperature and vapour pressure. Bioclimatic indices were calculated, which included relative humidity [34] and Humidex [35]. The total observation period covered the years 1992 till 2018, amounting to the processing of almost 175,000 weather observations.

Since Humidex often underestimates indoor discomfort and exposure at the workplace [36] it was not considered for this study, with average daily temperature used as the exposure temperature parameter as suggested by other studies [10, 20, 37, 38].

### Statistical analysis

#### Effects of ambient temperature

A time-series regression method was applied to examine the association between daily mean temperature and daily respiratory mortality. This was carried out using a distributed lag nonlinear model (DLNM) with a Poisson distribution [39]. Daily mean temperature was selected as the primary temperature variable as other studies have found it as a more suitable temperature indicator than minimum or maximum temperatures [10, 37, 40].

To explore every plausible exposure–response association, we estimated cold and heat effects on respiratory mortality at different temperature percentiles (i.e., 1st, 5th and 10th percentiles for cold effects; 90th, 95th and 99th percentiles for heat effects). Subgroup analysis was conducted to examine the effect of cold and hot temperatures across different demographic groups (i.e., age group and gender) and time periods.

To capture the non-linear lag-effects of temperature, a DLNM model was used with 4 degrees of freedom natural spline function for temperature, and 5 degrees of freedom natural cubic spline for a lag period up to 27 days. This is similar to the approach adopted in previous studies [41, 42]. The model was adjusted for covariates i.e., the flexible spline function of time (7 df/year) to control for the seasonal and long-term trends; day of the week (as dummy variables); holidays (as a binary variable, a holiday such as public holidays were coded 1); and the spline function of daily mean relative humidity with 4 degrees of freedom. We assumed a maximum lag of 27 days between exposure and deaths to allow for any harvesting effect [43, 44]. The harvesting effect refers to a phenomenon in which extreme temperatures can cause a sudden increase in the number of deaths in the first few days since exposure but then a decrease in the expected number of deaths for the next few days. The net result over a longer period (e.g., 27 days) can be zero in some cases. Thus, studies that only examine short lag periods will ignore this effect. The model can be outlined as follows,

$${\text{Log }}\left( {{\text{Y}}_{{\text{t}}} } \right) = \, \alpha \, + {\text{ cb }}\left( {{\text{Temp}}_{{{\text{t}},l,}} {\text{4df}}} \right) \, + {\text{ns }}\left( {{\text{RH}}_{{{\text{t}}, \, l}} ,{\text{ 4df}}} \right) \, + {\text{ ns }}\left( {{\text{time}},{ 7}*{\text{year}}} \right) \, + {\text{ DOW }} + {\text{ HO}} + {\text{ log}}\left( {{\text{Pop}}} \right)$$

where t refers to the day of the observation; Y_t_ is the observed daily respiratory death on day t; Temp_t,*l*_ is a matrix created by DLNM for daily mean temperature on day t for different lag days (0–27 days), where three internal knots are placed at equal spaces for the temperature variable and three internal knots are placed at equal intervals on the log scale for lag days; RH_t*,l*_ is the average humidity on day t; ns denotes the natural cubic spline function; DOW is the day of the week; HO is a holiday; Pop represents the standardised population (population on the log scale) to allow for changes in the population over time.

Estimates were generated as Relative Risks (RRs) with 95% confidence intervals and AIC estimates were used to examine the model’s goodness of fit.

#### Calculation of reference temperature (MMT)

A cross basis product was created for temperature and fitted into the DLNM model. We used the lowest AIC estimates for Poisson models to choose the degree of freedom for temperature and lag [39]. The model was adjusted for potential confounders. The reference temperature or minimum mortality temperature (MMT) was defined as the cut-off point temperature value associated with the lowest respiratory mortality risk. The estimated reference temperature was 22.1 °C for the study period.

### Sensitivity analysis

Sensitivity analysis was conducted to test the robustness of our results using a time stratified case-cross over analysis with strata defined as the days of the week in the same month of the same year to control for trends and seasonality [45].

The robustness of the used DLNM model was also checked using different knot placements (at 10th, 75th, and 90th percentile temperature), and adjusting for varying degrees of freedom for daily mean temperature (3–5 df).

Previously, many studies have reported a U-shaped relationship between temperatures and mortality [7, 8]. In this study, no significant heat impacts were observed for respiratory mortality with the DLNM model with Poisson distribution. Thus, as a part of the sensitivity analysis, a double threshold natural cubic spline DLNM model, which takes into account reference temperatures at cold and hot points, was also used to examine the consistency of estimates [45]. We have visually identified the two temperature thresholds (cold and hot temperature) from plots and examined the exposure–response relationship to confirm the observed cold effects below the cold threshold and capture if there is a heat effect above the hot threshold.

All statistical analysis was conducted using the R statistical software package (3.2.5) with the ‘dlnm’ package to fit regression models [39].

## Results

### Descriptive statistics

A total of 7679 deaths were recorded for respiratory illnesses during the year 1992–2017. The respiratory mortality rate was higher in males (57.94%) compared to females (42.06%). Respiratory deaths were mostly recorded for elderly people being ≥ 65 years in both sexes (Males: 90.70%; Females: 98.31%) compared to 16–64 years age group (Males: 8.81%; Females: 6.54%). Only (0.27%) of the respiratory mortality were listed for people ≤ 15 years of age and they were not included for further analysis.

**Fig. 1:**
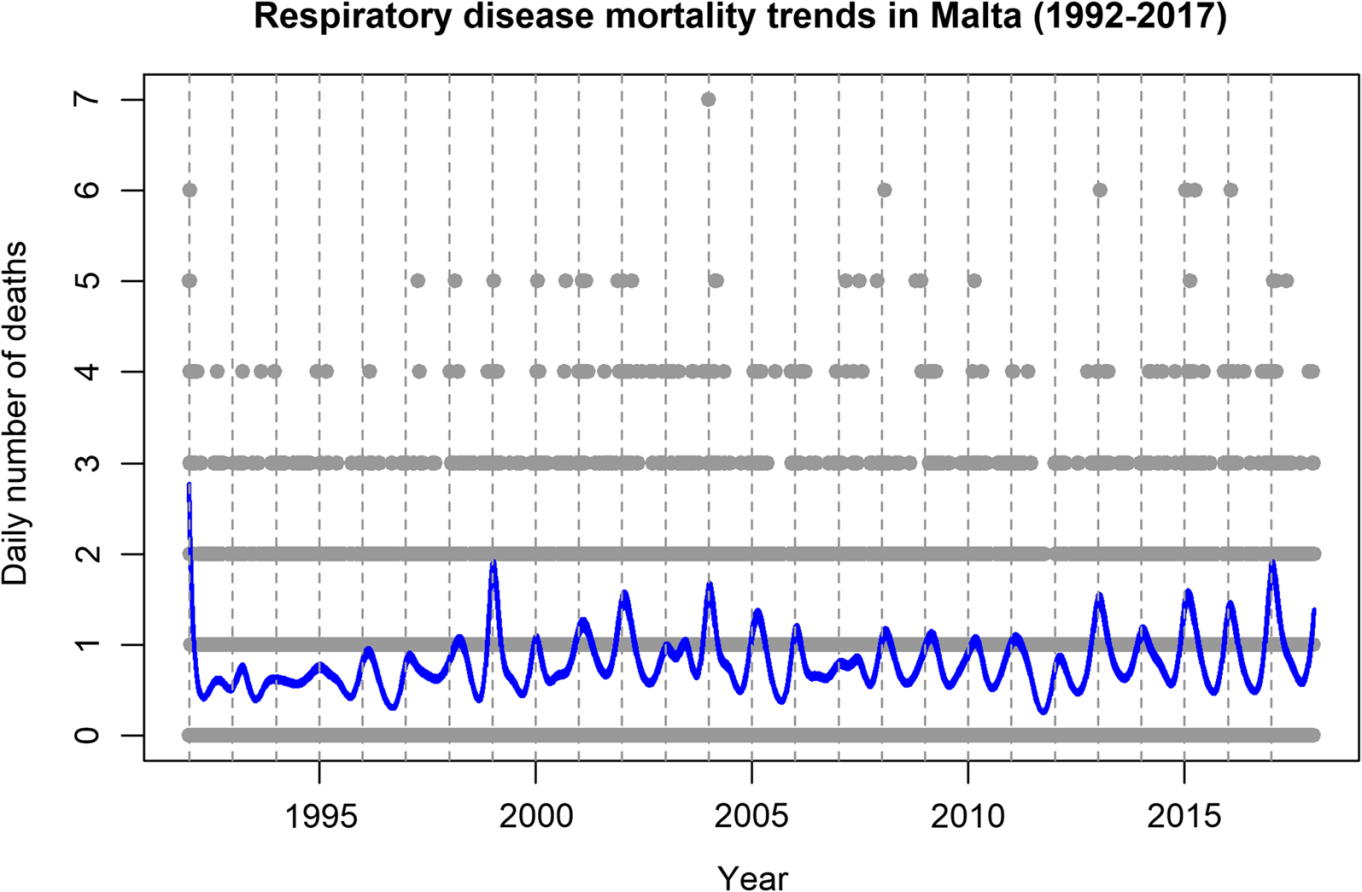
Daily number of respiratory deaths in Maltese population during 1992–2017 (The blue line indicates seasonality estimated from the regression model fitting a natural cubic spline function for time with 7 degrees of freedom)

Visually, the mortality trends in Malta showed a relatively consistent seasonal pattern during the study period (Fig. 1). Overall, the recorded deaths were higher in the coldest months (January–February) compared to the hottest months (June–August).

### Overall effect of extreme temperature on respiratory mortality

**Fig. 2:**
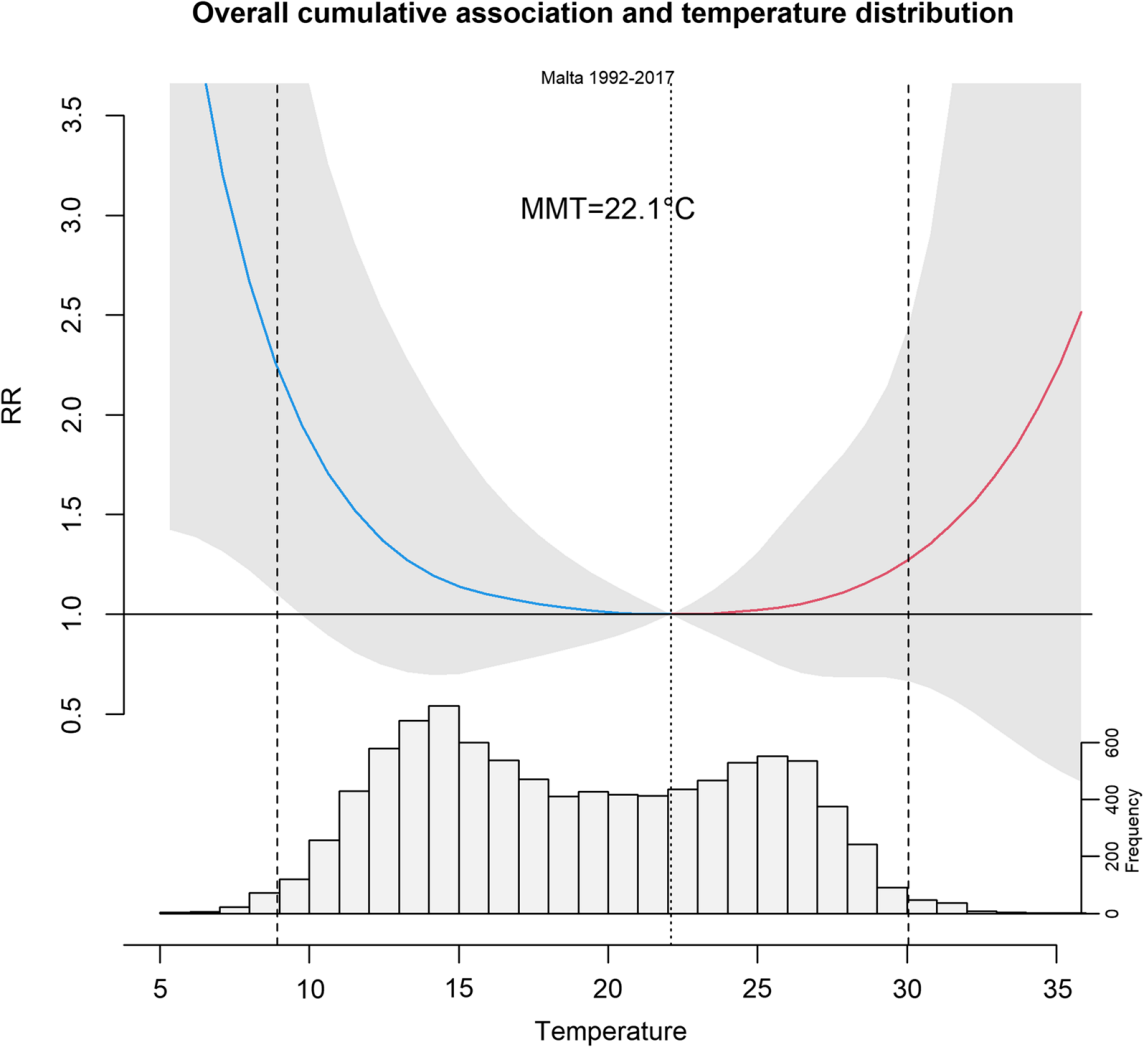
The estimated overall cumulative exposure–response association (RR) using MMT as the reference temperature

Figure 2 shows the temperature distribution and its overall effect on respiratory mortality. The effect of extreme temperatures was assessed as a Relative Risk (95%CI) using Minimum Mortality Temperature (MMT) as the reference temperature (22.1 °C).

Colder temperatures (8–15 °C) were consistently related to higher respiratory mortality. At 8.9 °C (1st percentile), the daily mean temperature was related to higher mortality due to respiratory illness (RR 2.24, 95%CI 1.10–4.54) (Table 1). However, higher temperatures (26.7–30.5 °C) did not show any increased effect (RR 1.3, 95%CI 0.75–2.25) on respiratory mortality.

**Table 1:** Overall relative risk estimates for cold effect and heat effect (at different temperatures)

Daily mean Temperature (percentiles)	Relative Risk	Lower	Upper
8.92 °C (1st)	2.24*	1.10*	4.54*
10.99 °C (5th)	1.62	0.86	3.08
12.06 °C (10th)	1.43	0.77	2.66
26.70 °C (90th)	1.06	0.70	1.61
27.86 °C (95th)	1.11	0.68	1.80
30.05 °C (99th)	1.28	0.67	2.44

Minimum mortality temperature (22.1 °C)

p-value * < 0.05, ** < 0.01, *** < 0.001

**Fig. 3:**
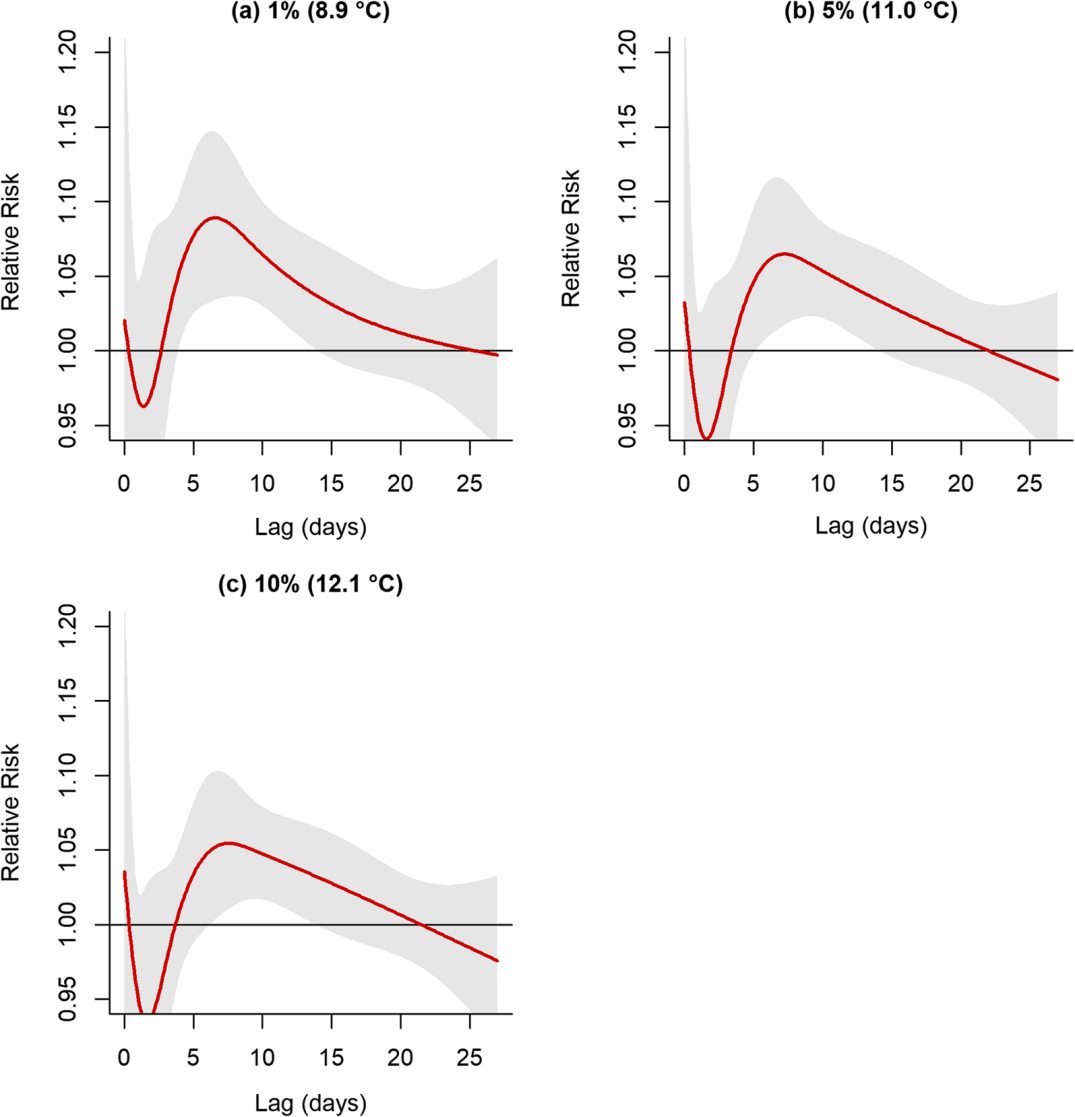
Shows the estimated relative risk of respiratory mortality by different cold temperatures (0–27 lag days)

Cold temperatures (8.9–12.1 °C) were consistently related to higher respiratory mortality over 4–14 days (Fig. 3). At 12.1 °C, daily mean temperature was related to higher mortality due to respiratory failure over 7–14 days (RR 1.06, 95%CI 1.02–1.10). At 11 °C, daily mean temperature increases the number of respiratory deaths over 6–14 days (RR 1.06, 95%CI 1.03–1.12). The risk for respiratory mortality increased at 8.9 °C temperature over 4–13 lag days (RR 1.09, 95%CI 1.04–1.15).

**Fig.4:**
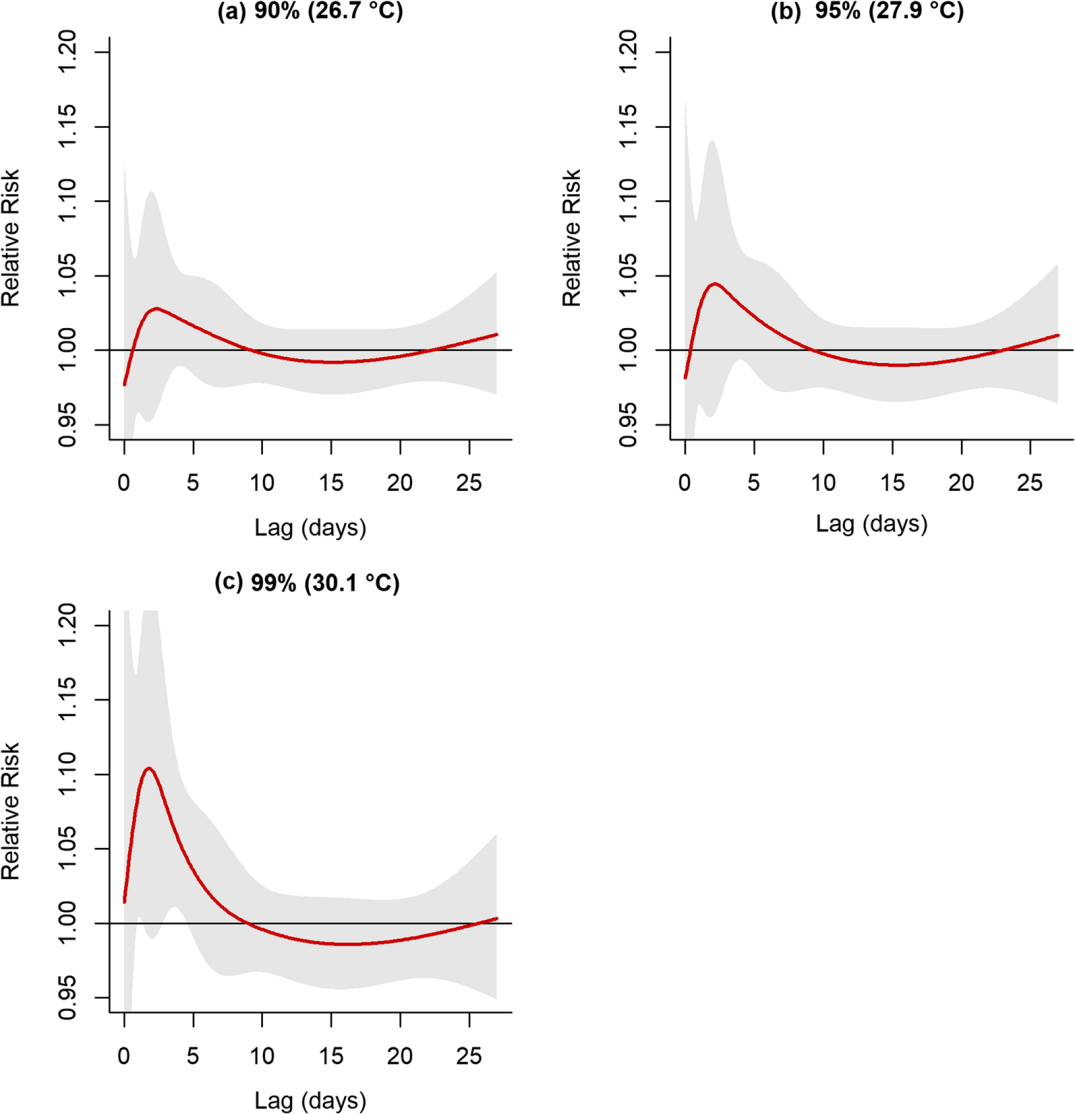
Shows the estimated relative risk of respiratory mortality by different hot temperatures (0–27 lag days)

There were no strong effects of hotter temperatures (90th percentile, 95th percentile) over the 0–27 lag days (Fig. 4). At 30.1 °C temperature (99th percentile), daily mean temperature was related to respiratory mortality at lag 4 (RR 1.06, 95%CI 1.01–1.11). However, this effect was inconsistent at other lag days.

### Subgroup analysis

The relative risk for respiratory mortality at different cold temperatures was higher for males (Table 2). In contrast, no effect of daily mean temperatures (both cold and hot) was observed for females. At colder temperatures (8.92–10.99 °C), the risk for respiratory mortality was higher in males (8.9 °C: RR 3.51, 95%CI 1.58–7.77; 10.99 °C: RR 2.03, 95%CI 1.06–3.90). The cold effect (8.92–10.99 °C) was similar for males across different age groups i.e., males Over 65 years (8.9 °C: RR 4.85, 95%CI 2.02–11.63) and males between 16 and 64 years (8.9 °C: RR 5.00, 95%CI 2.08–12.03). There was also no heat effect found for males across different age groups.

**Table 2:** Estimated Relative Risk (RR) for respiratory mortality by different temperatures in different groups

Temperature (°C) Percentiles	Male RR (95%CI)	Female RR (95%CI)	Male > 65 years RR (95%CI)	Female > 65 years RR (95%CI)	Male 16–64 years RR (95%CI)	Female 16–64 years. RR (95%CI)
8.92 °C (1st)	3.51 (1.58–7.77) *	2.13 (0.51–8.93)	4.85 (2.02–11.63) *	1.75 (0.40–7.60)	5.00 (2.08–12.03) *	1.78 (0.41–7.70)
10.99 °C (5th)	2.03 (1.06–3.90)*	2.17 (0.57–8.32)	2.60 (1.23–5.52)*	1.75 (0.44–6.89)	2.68 (1.23–5.71)*	1.77 (0.45–6.98)
12.06 °C (10th)	1.63 (0.88–3.01)	2.17 (0.58–8.12)	2.02 (0.98–4.13)	1.73 (0.45–6.67)	2.07 (1.00–4.26)*	1.75 (0.46–6.75)
26.70 °C (90th)	1.57 (0.73–3.42)	1.01 (0.95–1.06)	1.56 (0.74–3.32)	1.00 (0.97–1.03)	1.54 (0.73–3.23)	1.00 (0.97–1.03)
27.86 °C (95th)	1.69 (0.72–3.97)	1.02 (0.90–1.15)	1.67 (0.72–3.86)	1.02 (0.88–1.20)	1.63 (0.71–3.74)	1.03 (0.88–1.20)
30.05 °C (99th)	1.77 (0.65–4.80)	1.36 (0.67–2.74)	1.65 (0.60–4.54)	1.29 (0.61–2.75)	1.58 (0.58–4.33)	1.31 (0.61–2.78)

Minimum mortality temperature (22.1 °C)

p-value * < 0.05, ** < 0.01, *** < 0.001

### Effect of cold and hot temperatures over periods

**Fig. 5:**
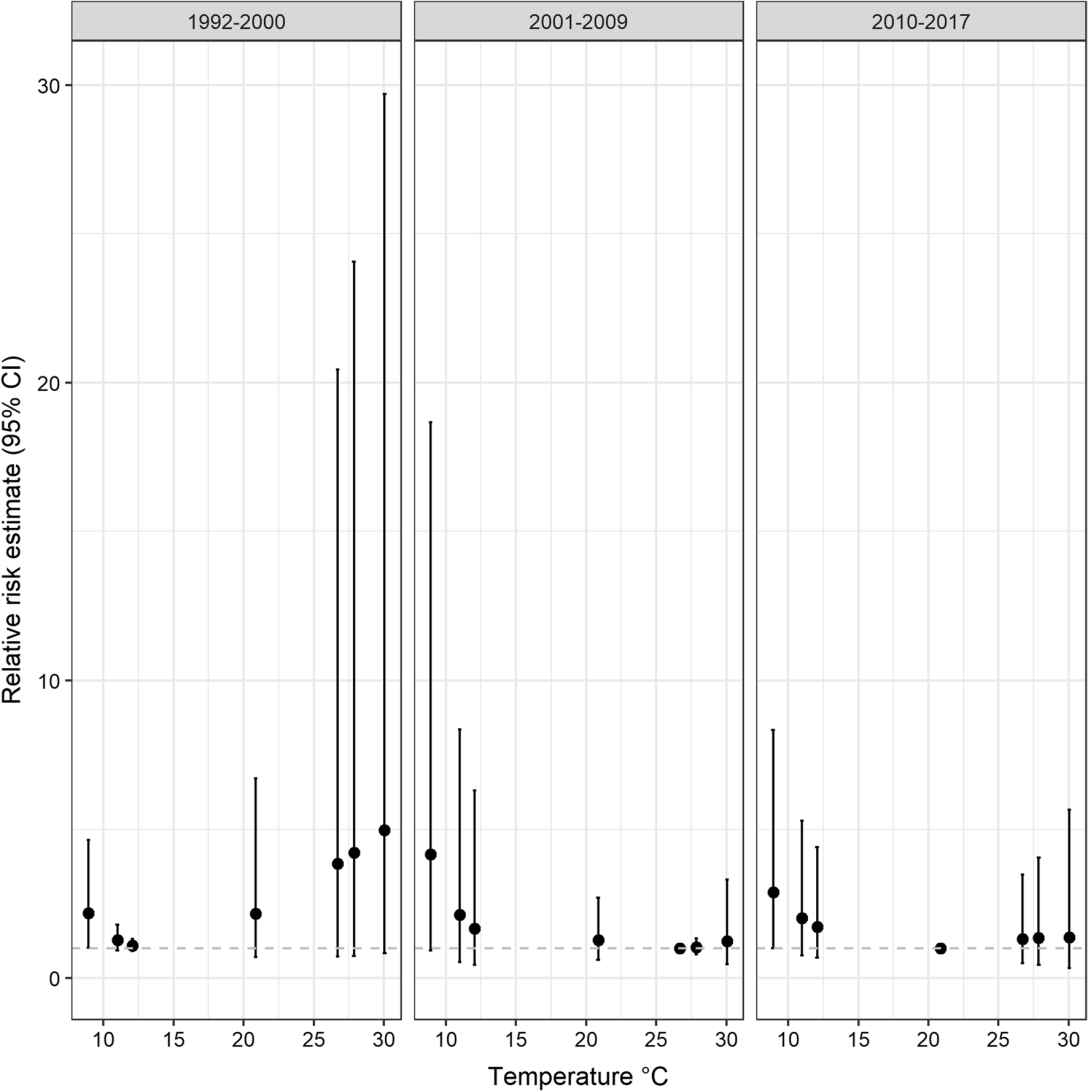
Shows the estimated relative risk of respiratory mortality for different temperatures across different time periods

Cold temperature was related to respiratory deaths in the earliest time period (1992–2000) (Fig. 5). During this time period, cold temperature (at 8.9 °C) increased the risk for respiratory mortalities (RR 2.18, 95%CI 1.02–4.64). In contrast, the effect of cold temperature (8.9 °C) on respiratory mortality was not related for the later time periods, 2001–2009 (8.92 °C: RR 4.17, 95%CI 0.93–18.67) and 2010–2017 (RR 2.89, 95%CI, 1.00–8.34). However, no heat effect (26.70–30.05 °C) was observed during these time periods.

### Sensitivity analysis

The estimated relative risks for respiratory mortality by the case-crossover analysis were similar to the results obtained from the DLNM model (See Additional file 1). The DLNM findings for the non-linear lag-effects of temperature with manually placed knots for daily mean temperature (10th, 75th, and 90th percentile) remain the same as the reported findings (See Additional file 2). The varying degree of freedom for temperature (3–5 df) also didn’t show any noticeable changes in the relationships between temperature and respiratory mortality.

We also used a double threshold model to allow for modelling of cold and hot threshold temperatures at the same time and observed similar findings (i.e., increased risks from cold effects and no increase in risk for heat effects) to those already presented (See Additional file 3).

## Discussion

Overall, our study found that daily mean temperature, specifically colder temperature, was associated with respiratory deaths in Malta during the period from 1992 to 2017. In this study, colder temperatures (8–15 °C) increase the risk for respiratory mortality (RR 2.24, 95%CI 1.10–4.54). However, no relationship was found between hot temperatures (26.70–30.05 °C) and daily respiratory deaths (RR 1.3, 95%CI 0.75–2.25). While most of the previous studies from Europe found a J or U-shaped association between daily mean temperature and respiratory mortality [7, 8, 11, 12, 17, 46], a similar association was not found for the Maltese Islands. Analitis et al. [11] examined the effect of cold weather on cause-specific mortality in 15 European countries and reported a 1 °C decrease in the temperature was associated with the highest increase in the daily number of deaths due to respiratory causes (3.30%, 95%CI 2.61–3.99%).

Contradicting previous European studies, the study observed no increase in risk of respiratory mortality related to heat effects, although we did observe quite a lot of uncertainty around this effect with wide 95% CI estimates. This could be due a lower number of daily death counts during the study period. While the cold weather in Malta is relatively mild compared to Nordic Europe, a greater number of deaths were recorded during the winter months compared to the summer months. This could be plausible as the local population may be more adapted to hot weather than cold weather [29], especially in Mediterranean countries due to different levels of public health intervention [45]. Fowler et al. [47] conducted a multi-country analysis and reported a higher winter mortality risk for Malta compared to the Scandinavian countries that experienced the coldest winters in Europe. In Malta, there has also been a significant increase in the use of air conditioning over the last two decades which could help to explain no clear heat effect on respiratory mortality [48]. Healy [49] conducted a cross country analysis of excess winter mortality in European countries and suggested that countries experiencing greater inter–annual variability in winter temperatures (and may not be lowest winter temperatures) have greater excess winter mortality compared to countries with colder winters.

In this study, we found an increased effect of colder temperatures on respiratory deaths over 4–14 lag days. At 8.9–12.1 °C (1st percentile–10th percentile), the risk for daily mortality due to respiratory illness increased by 6–9% (95%CI 1.02–1.15). Most studies from Europe reported that higher temperatures had an adverse effect on cardio-respiratory mortality over 0–1 and 27 lag days which dropped significantly in the following days 8–14 days and 15–21 days [8, 9, 46]. Analitis et al. [11] conducted a multi-city analysis and reported that the effect of cold temperatures was greater in warmer (southern) climates and could persist up to 0–23 days. Only one study was found from Malta [29] that assessed temperature effect on all-cause mortality across seasons after adjusting for a lag effect for 0–15 days and reported a higher risk for daily deaths during summer months. Due to lack of evidence from Malta, the chance of a direct comparison of temperature effects on respiratory mortality was limited. However, it was clear that cold effects on mortality could persist for a long time (even greater than 60 days) compared to a heat effect [50].

Stratified analysis in this study found a significant effect of daily mean cold temperatures on daily respiratory deaths among males of 16–64 years age group and over 65 years age group. Previous studies have reported that elderly people (over 65 years) can be more affected by extreme temperatures compared to younger people (16–64 years) [10, 11, 29, 50]. Most of these studies estimated the risk in different age groups but didn’t examine differences between males and females. In this study we found that the risk for daily respiratory mortality was higher for males of 16–64 years age groups (RR 5.00, 95%CI 2.08–12.03) and also for males over 65 years (RR 4.85, 95%CI 2.2–11.63) at 8.9 °C (1st percentile). However, no effect of daily mean temperature on respiratory mortality was observed in females of different age groups (i.e., 16–64 years, > 65 years).

In addition, the analysis of cold and hot effects over different time periods shows that colder temperatures were significantly related to higher respiratory mortality rate during the year 1992–2000. At 8.9 °C, the risk for respiratory deaths were higher during the year 1992–2000 (RR 2.18, 95%CI 1.02–4.64). However, no increased risk for respiratory mortality due to cold temperature was observed for the time periods 2001–2009 and 2010–2017. This change in the estimated relative risks over time could be due to changes in adaptive strategies [51]. As discussed earlier, in countries with a milder winter, there could be the possibility that as people become more adapted to hot weather, they are less adapted to colder temperatures [29, 49]. Moreover, countries with wide variability in the inter-annual mean winter temperature compared to those with consistent year-to-year winter temperature could experience a higher number of winter mortality as a human thermoregulatory system, behaviour and adaptation strategies (i.e., protective clothing, insulted housing) might be less developed [47, 52]. Due to climate change impacts (i.e., rise in the overall daily mean temperature), people may be experiencing more inter–annual variability in winter temperatures for the last two decades and gradually improved their preventative and mitigating strategies, which possibly decreased the risk in later years.

The study has some important strengths. First, to the best of our knowledge, this is the first study from Malta to examine the effect of ambient temperature specifically for respiratory deaths. The findings provide good evidence to confirm the adverse effect of cold temperatures on respiratory mortality across different time periods, age groups and gender. Second, this study utilized a high quality and accurate dataset comprised of a relatively large sample size and long-time series of meteorological data from Malta. This provides a relatively high degree of statistical power and confidence to establish associations. Third, a range of sophisticated and complex statistical models were employed in order to provide robust and accurate results for a better understanding of the non-linear exposure–response relationships and lag effect (0–27 lag days) of daily mean temperature on respiratory mortality.

Some limitations should be mentioned. First, the meteorological data were only collected from the only official climatological station in Malta, so it was difficult to estimate the degree of site-specific exposure. However, Malta is a densely populated small country, so the chances of estimation bias is minimal. Secondly, the number of deaths due to respiratory illnesses could be underestimated due to changes in the ICD-10 coding after the year 2008. For example, patients who died with a chest infection but also had dementia would be recorded as deaths due to dementia. Thirdly, while this study provided some statistical evidence on the relationship between cold temperatures and respiratory deaths, there could be other factors such as seasonal influenza, history of COPD, level of exposure (i.e., housing type, indoor temperature, coping measures, air quality) and physical comorbidities which could be considered in the analysis for a better understanding of the relationship. For example, some studies have previously reported that seasonal influenza during winter periods could trigger respiratory complications [53, 54].

## Conclusions

The study provides good evidence of cold effects on respiratory mortality. However, the temperature effect on respiratory mortality could be greatly influenced by other potentially interacting, individual and environmental factors. In this regard, some physical and sociodemographic characteristics need further exploration in virtue of the observed climate change patterns for the Maltese Islands. Public awareness, preventive measures (i.e., adaptation strategies) and timely intervention may be needed in order to prevent the effect of inter-annual temperature variability on respiratory mortality. Policymakers should consider the impact of moderately cold temperatures on human health and take necessary action to reduce cold exposure and mitigate the mortality risk as part of current preventive measures and strategies particularly for vulnerable populations.

## Supplementary Information

**Additional file 1: Fig. S1** the estimated overall cumulative exposure–response association (RR) using a case-crossover approach.

**Additional file 2: Fig. S2** the estimated overall cumulative exposure–response association (RR) using manually placed knots for temperature.

**Additional file 3: Fig. S3** the estimated relative risk of temperature-related respiratory mortality using a double threshold model.

## Data Availability

The data that support the findings of this study are available on request from the corresponding author.

## Code availability

Available on request.
