# Supplementary figures and images for: The adverse effect of ambient temperature on respiratory deaths in a high population density area: the case of Malta
Source: Respir Res. 2022 Oct 31;23:299. doi: 10.1186/s12931-022-02218-z (PMC9623984; doi:10.1186/s12931-022-02218-z)

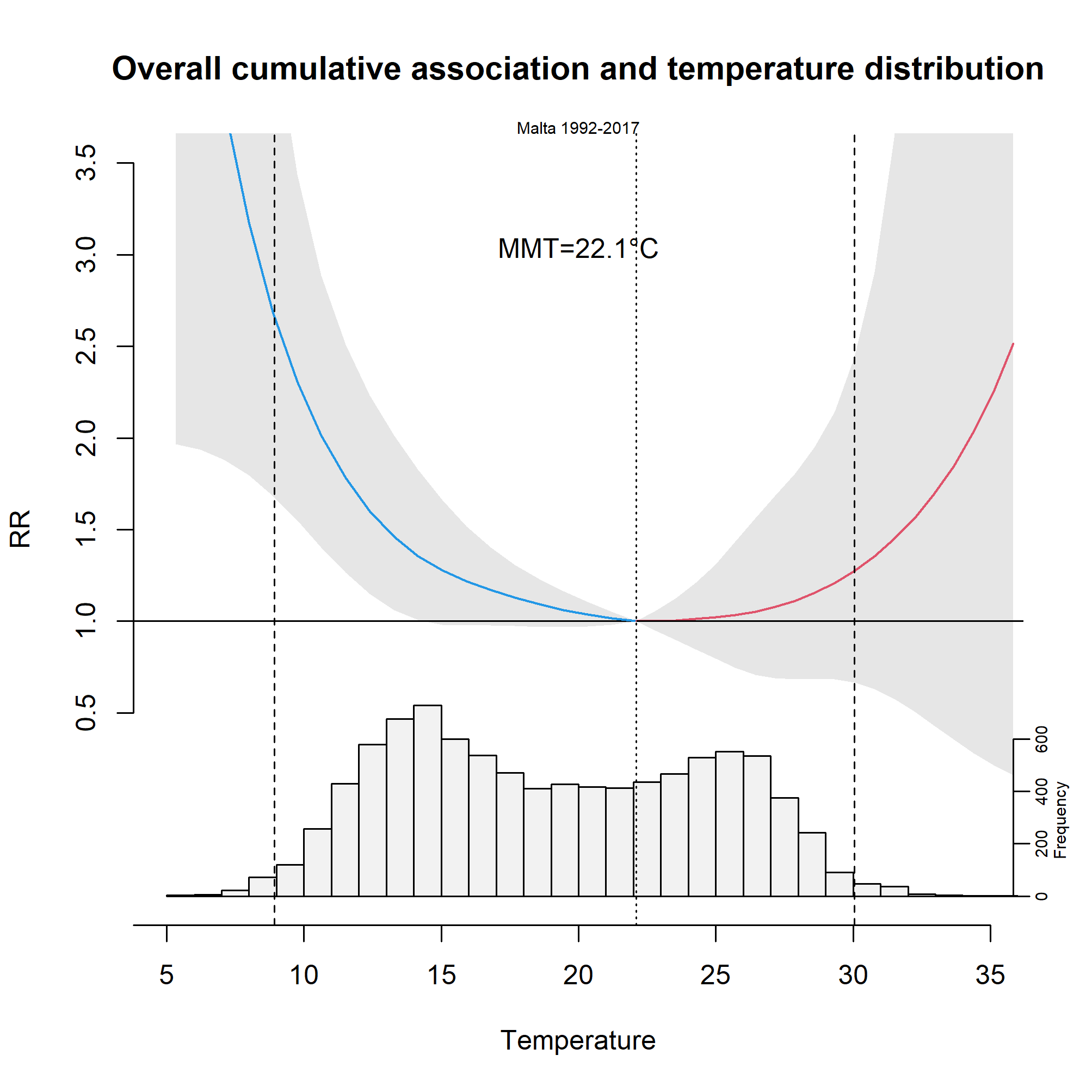

Supplement: Supplementary file 1 — Additional file 1: Fig. S1 the estimated overall cumulative exposure–response association (RR) using a case-crossover approach. [file 12931_2022_2218_MOESM1_ESM.tif]

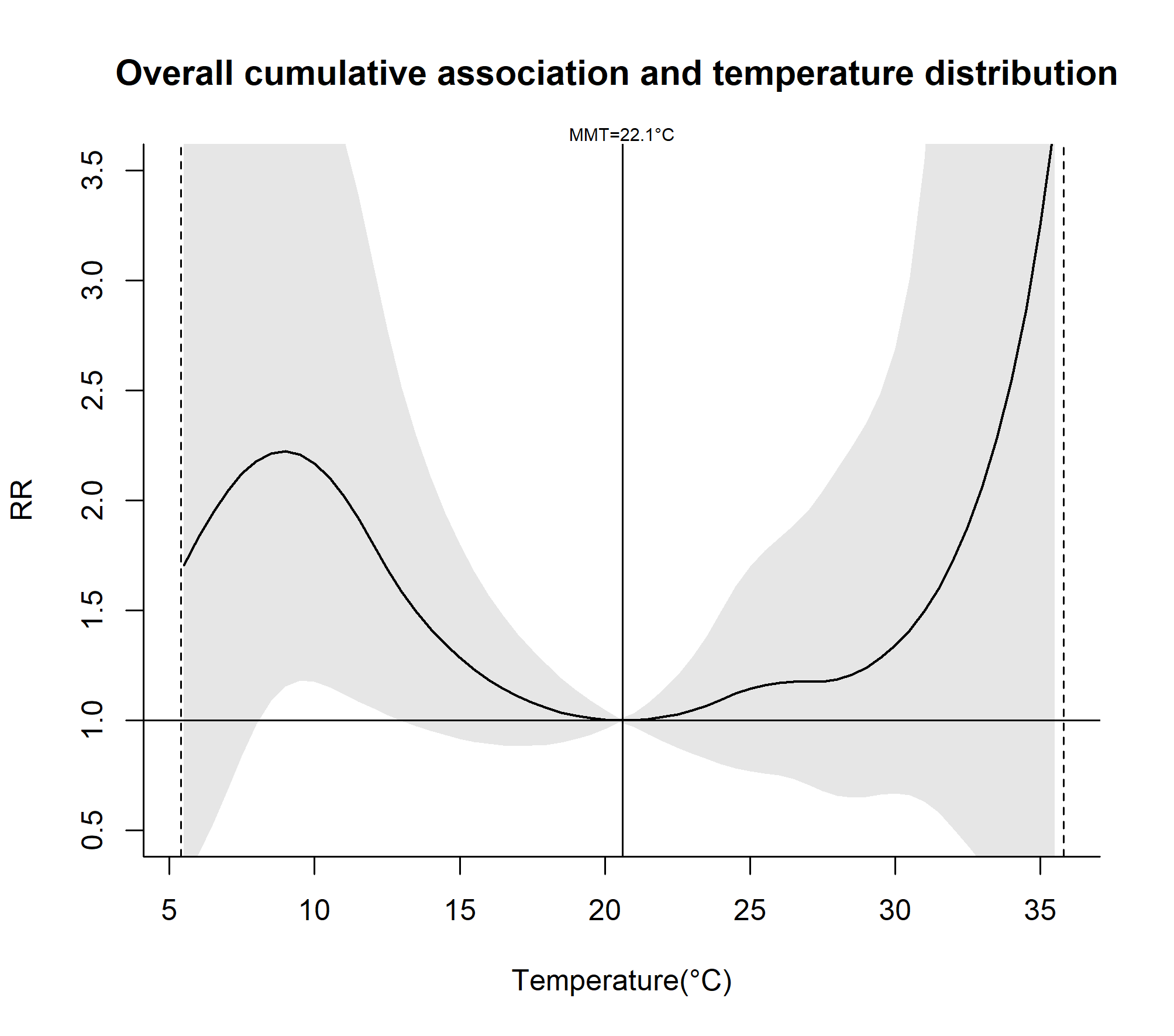

Supplement: Supplementary file 2 — Additional file 2: Fig. S2 the estimated overall cumulative exposure–response association (RR) using manually placed knots for temperature. [file 12931_2022_2218_MOESM2_ESM.tiff]

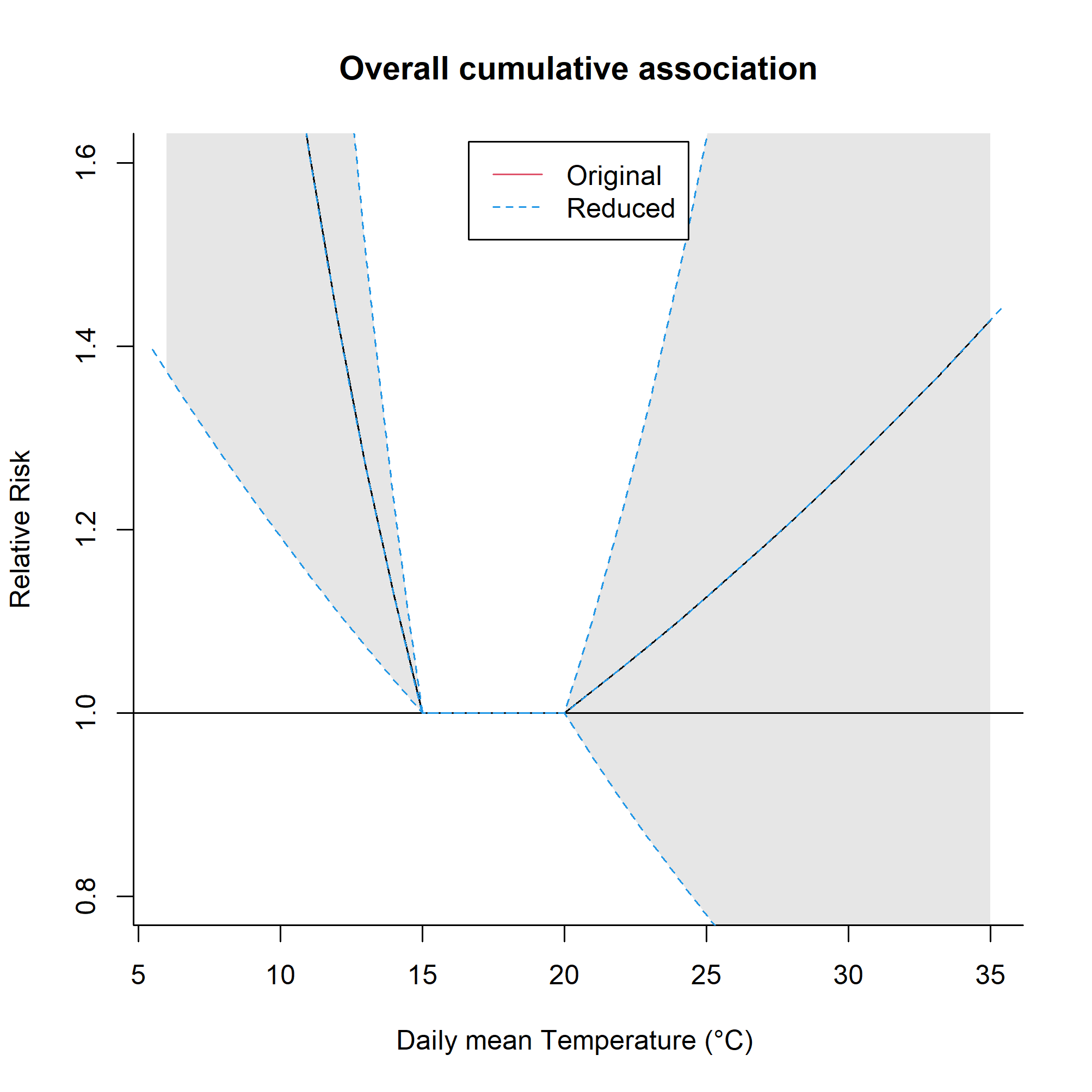

Supplement: Supplementary file 3 — Additional file 3: Fig. S3 the estimated relative risk of temperature-related respiratory mortality using a double threshold model. [file 12931_2022_2218_MOESM3_ESM.tiff]
